# Pangenomics and single-cell transcriptomics uncover the genetic basis of continuous bearing trait in grapevine

**DOI:** 10.1093/hr/uhaf228

**Published:** 2025-09-02

**Authors:** Yuting Liu, Yang Dong, Xinyue Fang, Xu Wang, Jiacui Li, Tianhao Zhang, Qiming Long, Ying Su, Xiaoya Shi, Xiangnian Su, Yingchun Zhang, Ting Hou, Mengrui Du, Yiran Ren, Peipei Wang, Xinglong Ji, Yunjun Chang, Sheng Yan, Sifan Yang, Yongfeng Zhou, Yanling Peng, Xiangpeng Leng

**Affiliations:** College of Horticulture, Qingdao Agricultural University, Qingdao 266109, China; National Key Laboratory of Tropical Crop Breeding, Shenzhen Branch, Guangdong Laboratory of Lingnan Modern Agriculture, Key Laboratory of Synthetic Biology, Ministry of Agriculture and Rural Affairs, Agricultural Genomics Institute at Shenzhen, Chinese Academy of Agricultural Sciences, Shenzhen, China; National Key Laboratory of Tropical Crop Breeding, Shenzhen Branch, Guangdong Laboratory of Lingnan Modern Agriculture, Key Laboratory of Synthetic Biology, Ministry of Agriculture and Rural Affairs, Agricultural Genomics Institute at Shenzhen, Chinese Academy of Agricultural Sciences, Shenzhen, China; National Key Laboratory of Tropical Crop Breeding, Shenzhen Branch, Guangdong Laboratory of Lingnan Modern Agriculture, Key Laboratory of Synthetic Biology, Ministry of Agriculture and Rural Affairs, Agricultural Genomics Institute at Shenzhen, Chinese Academy of Agricultural Sciences, Shenzhen, China; National Key Laboratory of Tropical Crop Breeding, Shenzhen Branch, Guangdong Laboratory of Lingnan Modern Agriculture, Key Laboratory of Synthetic Biology, Ministry of Agriculture and Rural Affairs, Agricultural Genomics Institute at Shenzhen, Chinese Academy of Agricultural Sciences, Shenzhen, China; National Key Laboratory of Tropical Crop Breeding, Shenzhen Branch, Guangdong Laboratory of Lingnan Modern Agriculture, Key Laboratory of Synthetic Biology, Ministry of Agriculture and Rural Affairs, Agricultural Genomics Institute at Shenzhen, Chinese Academy of Agricultural Sciences, Shenzhen, China; National Key Laboratory of Tropical Crop Breeding, Shenzhen Branch, Guangdong Laboratory of Lingnan Modern Agriculture, Key Laboratory of Synthetic Biology, Ministry of Agriculture and Rural Affairs, Agricultural Genomics Institute at Shenzhen, Chinese Academy of Agricultural Sciences, Shenzhen, China; National Key Laboratory of Tropical Crop Breeding, Shenzhen Branch, Guangdong Laboratory of Lingnan Modern Agriculture, Key Laboratory of Synthetic Biology, Ministry of Agriculture and Rural Affairs, Agricultural Genomics Institute at Shenzhen, Chinese Academy of Agricultural Sciences, Shenzhen, China; National Key Laboratory of Tropical Crop Breeding, Shenzhen Branch, Guangdong Laboratory of Lingnan Modern Agriculture, Key Laboratory of Synthetic Biology, Ministry of Agriculture and Rural Affairs, Agricultural Genomics Institute at Shenzhen, Chinese Academy of Agricultural Sciences, Shenzhen, China; National Key Laboratory of Tropical Crop Breeding, Shenzhen Branch, Guangdong Laboratory of Lingnan Modern Agriculture, Key Laboratory of Synthetic Biology, Ministry of Agriculture and Rural Affairs, Agricultural Genomics Institute at Shenzhen, Chinese Academy of Agricultural Sciences, Shenzhen, China; National Key Laboratory of Tropical Crop Breeding, Shenzhen Branch, Guangdong Laboratory of Lingnan Modern Agriculture, Key Laboratory of Synthetic Biology, Ministry of Agriculture and Rural Affairs, Agricultural Genomics Institute at Shenzhen, Chinese Academy of Agricultural Sciences, Shenzhen, China; National Key Laboratory of Tropical Crop Breeding, Shenzhen Branch, Guangdong Laboratory of Lingnan Modern Agriculture, Key Laboratory of Synthetic Biology, Ministry of Agriculture and Rural Affairs, Agricultural Genomics Institute at Shenzhen, Chinese Academy of Agricultural Sciences, Shenzhen, China; College of Horticulture, Qingdao Agricultural University, Qingdao 266109, China; National Key Laboratory of Tropical Crop Breeding, Shenzhen Branch, Guangdong Laboratory of Lingnan Modern Agriculture, Key Laboratory of Synthetic Biology, Ministry of Agriculture and Rural Affairs, Agricultural Genomics Institute at Shenzhen, Chinese Academy of Agricultural Sciences, Shenzhen, China; National Key Laboratory of Tropical Crop Breeding, Shenzhen Branch, Guangdong Laboratory of Lingnan Modern Agriculture, Key Laboratory of Synthetic Biology, Ministry of Agriculture and Rural Affairs, Agricultural Genomics Institute at Shenzhen, Chinese Academy of Agricultural Sciences, Shenzhen, China; College of Horticulture, Qingdao Agricultural University, Qingdao 266109, China; College of Horticulture, Qingdao Agricultural University, Qingdao 266109, China; College of Horticulture, Qingdao Agricultural University, Qingdao 266109, China; Shandong Fresh Table Grape Research Institute, Qingdao, China; College of Horticulture, Qingdao Agricultural University, Qingdao 266109, China; National Key Laboratory of Tropical Crop Breeding, Shenzhen Branch, Guangdong Laboratory of Lingnan Modern Agriculture, Key Laboratory of Synthetic Biology, Ministry of Agriculture and Rural Affairs, Agricultural Genomics Institute at Shenzhen, Chinese Academy of Agricultural Sciences, Shenzhen, China; College of Horticulture, Qingdao Agricultural University, Qingdao 266109, China; National Key Laboratory of Tropical Crop Breeding, Shenzhen Branch, Guangdong Laboratory of Lingnan Modern Agriculture, Key Laboratory of Synthetic Biology, Ministry of Agriculture and Rural Affairs, Agricultural Genomics Institute at Shenzhen, Chinese Academy of Agricultural Sciences, Shenzhen, China; National Key Laboratory of Tropical Crop Breeding, Shenzhen Branch, Guangdong Laboratory of Lingnan Modern Agriculture, Key Laboratory of Synthetic Biology, Ministry of Agriculture and Rural Affairs, Agricultural Genomics Institute at Shenzhen, Chinese Academy of Agricultural Sciences, Shenzhen, China; National Key Laboratory of Tropical Crop Breeding, Tropical Crops Genetic Resources Institute, Chinese Academy of Tropical Agricultural Sciences, Haikou, China; National Key Laboratory of Tropical Crop Breeding, Shenzhen Branch, Guangdong Laboratory of Lingnan Modern Agriculture, Key Laboratory of Synthetic Biology, Ministry of Agriculture and Rural Affairs, Agricultural Genomics Institute at Shenzhen, Chinese Academy of Agricultural Sciences, Shenzhen, China; College of Horticulture, Qingdao Agricultural University, Qingdao 266109, China

## Abstract

Grapevine (*Vitis vinifera*) is economically important for fresh consumption, winemaking, and drying. The circadian systems of flowering and fruit development are crucial for viticulture and yield formation. However, the genetic basis of continuous flowering and bearing has been rarely elucidated. Here, we integrate pan-genomics, comparative genomics, single-cell transcriptomics, and bulk transcriptomics to investigate the continuous flowering and bearing trait (CFB) in the ‘Julian’ grape, which bears fruits at different development stages from flower to mature berries simultaneously. Pan-genomics and comparative genomics discovered 558 unique structural variations (SVs) and eight genes enriched in flowering pathways exclusively in Julian, based on the newly generated haplotype-resolved near telomere-to-telomere (T2T) genome of ‘Julian’ and 15 previously published genomes of grapevine, which bears fruits in the same developmental stage (i.e., seasonal flowering and bearing, SFB). Single-cell transcriptomes of flowering buds for CFB ‘Julian’ and SFB ‘Muscat Hamburg’ detected seven distinct cell types, which provide detailed cell-type-specific gene expression profiles for both cultivars, with differential gene expression (DEG) insights highlighting growth, metabolism, and hormonal regulation pathways in ‘Julian’. Integrating SVs and DEG data, we pinpoint 37 candidate genes potentially associated with CFB, including Auxin/IAA, Cytochrome P450, Vicilin-like antimicrobial peptides (*AMP*) and respiratory burst oxidase homolog protein B (*RBOH*) related genes. This study provides insights into the genetic basis of CFB in grapevine, facilitating grapevine breeding with continuous flowering and bearing.

## Introduction

The grapevine is one of the most widely cultivated deciduous fruit trees, with significant economic value, primarily for fresh consumption, winemaking, and drying [1]. In the breeding process, the ability of the plant to form flowers is a key indicator of its potential for producing new varieties, and good bud differentiation is the basis for obtaining a considerable yield and quality of grapes. Furthermore, flower formation ability is a quantitatively inherited trait, which is influenced by environmental factors and multiple genes [2, 3]. Most grape cultivars exhibit flowering and fruiting within a concentrated period, known as seasonal flowering and bearing (SFB), while the grape cultivar ‘Julian’ (JA) is notable for its continuous flowering and bearing (CFB), which offers significant advantages for agricultural production, including an extended harvest period, increased yield, and improved resource utilization throughout the growing season. Beyond grapevines, CFB is also found in other economically important plants, such as day-neutral or everbearing strawberries [4], indeterminate tomatoes [5], and certain varieties of roses [6], figs. [7], and sweet pepper [8]. Although these traits are critical for agricultural production, most studies have focused on the physiological aspects of CFB in these crops, such as flowering timing and resource allocation [9, 10]. Previous studies on CFB have relied on single-omic approaches, such as identifying key regulators like *TERMINAL FLOWER 1* (*TFL1*) in roses and strawberries [11]. However, the dissection of genetic basis using single-omic is largely limited because its incomplete information. By contrast, integrated multi-omics, especially the integration of pan-genome, comparative genome, single-cell, and bulk transcriptomes, provides a chance to uncover the genetic basis of CFB comprehensively and precisely.

Pan-genome can uncover rare or specific genes that may not be present in a single reference genome, and these genes can be crucial under specific conditions [12]. A tomato pan-genome revealed rare alleles linked to fruit flavor, offering new possibilities for improving crop quality [13]. In rice, pan-genome studies uncovered large deletions, such as those affecting OsWAK112d, which enhance disease resistance and environmental adaptation [14]. Similarly, in sunflower, pan-genomes identified introgressed biotic resistance genes, enriching the genetic diversity of cultivated lines [15]. The application of the pan-genome in grapevine has enabled the identification of SVs/genes associated with 29 agronomic traits [16] and 7% of unique genes to each accession [17]. Similar to pan-genome, comparative genome studies have revealed the genetic basis of seedlessness in grapevine [18]. Up to now, such an analysis had not been conducted on the dissection of the genetic basis for CFB. Nevertheless, these examples demonstrate the power of the pan-genome and comparative genome analysis to uncover critical variations associated with traits like CFB in grapevine in JA, providing valuable insights for breeding strategies.

The advantage of single-cell transcriptomics has enabled the characterization of the transcriptome in hundreds of thousands of individual cells, providing unique insights into gene expression, cellular heterogeneity, and dynamic cellular differentiation trajectories, which are often obscured in bulk transcriptomics [19–21]. This technology has been widely applied across various plant species, including model organisms and economically important crops such as Arabidopsis [22, 23], rice [24], tomato [25], maize [26], and litchi [27]. For instance, single-cell transcriptional profiles of etiolated, de-etiolating, and light-grown *Arabidopsis thaliana* seedlings furnished information about the light signaling network at the cellular level and enhanced our comprehension of how light regulates plant development at the cellular and genome-wide levels [28]. Similarly, the single-cell transcriptome of the stem tip meristematic tissue cells in tomato generated high-resolution expression profiles and distinguished cell types [25]. Furthermore, single-cell transcriptomes are able to identify genes associated with flower-forming transformation in lychee using apical buds at different developmental stages [27]. Thus, the application of single-cell transcriptomes in grape flower bud is able to identify cell heterozygosity and detect lowly expressed genes at unprecedented resolution, and then help understand the contribution of genes to continuous flowering and fruiting of JA.

In this study, integrative analysis of multi-omics, including pan-genomics, comparative genomics, single-cell, and bulk transcriptomics was used to elucidate the genetic basis underlying CFB in this JA grape. Firstly, we newly assembled the T2T genomes of JA and performed comparative genome analysis with ‘Muscat Hamburg’ (MH) and PN40024. Secondly, we constructed a graph-based pan-genome using the newly assembled genomes and 15 previously published chromosome-level grape genomes, which produce flowers and fruit at a concentrated time (i.e., seasonal flowering and bearing, SFB). The pan-genomic and comparative genomic studies enabled us to characterize the unique variations and genes present in JA grape that may be correlated with CFB. Thirdly, we constructed high-resolution single-cell gene expression profiles for JA and MH, with a total of 13 cell clusters, and 7 cell types identified. Differentially expressed genes (DEGs) in the two cultivars were compared across the different cell clusters. Finally, we integrated pan-genomics, comparative genomics, single-cell, and bulk transcriptomics to screen a set of key candidate genes and SVs that may be involved in regulating the floral transition in JA. These findings from the present integrative genomic analysis will advance grapevine genomic breeding for CFB.

## Results

### Haplotype-resolved genome assembly and annotation of JA

**Figure 1:**
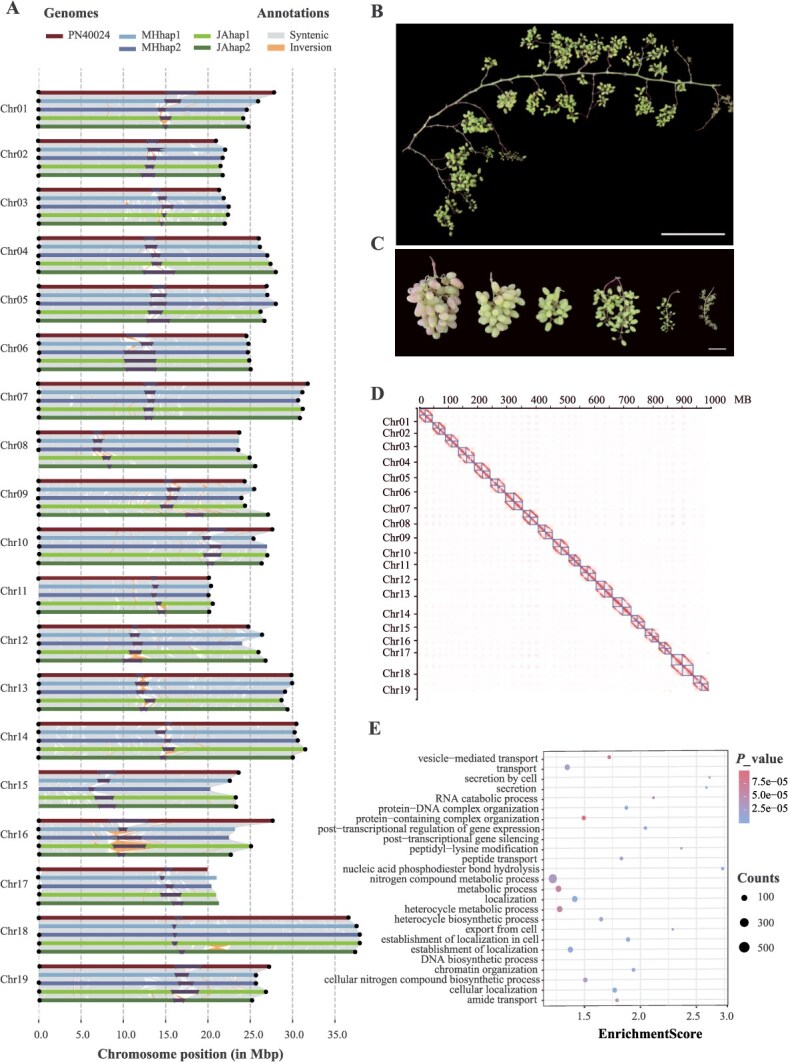
Genome assembly and analysis of the ‘Julian’ grape cultivar. (A) Synteny analysis comparing the haplotype-resolved ‘Julian’ genome (JA_hap1 and JA_hap2) with the reference genome PN40024 and ‘Muscat Hamburg’ (MH_hap1 and MH_hap2), displaying conserved genomic regions. (B, C) Morphological representation of JA phenotypes, including inflorescences and mature fruits at different node positions. Scale bars: 50 cm in (B) and 5 cm in (C). (D) Hi-C contact heatmap displaying the chromosomal interactions for the 19 pseudo-chromosomes of JA supporting the structural accuracy of the assembled genome. (E) GO enrichment analysis for hemizygous genes in JA, showing enrichment in biological processes.

To investigate the genetic basis of CFB in the JA cultivar (Fig. 1B and C), we generated high-quality haplotype-resolved genome assemblies (JA_hap1 and JA_hap2) using ~26.23 Gb PacBio HiFi sequencing (~66× coverage) and ~ 29.59 Gb Hi-C sequencing (~38.49× coverage). K-mer analysis estimated a heterozygosity rate of 1.44% for the JA genome, comparable to seedless cultivars, such as ‘Thompson Seedless’ (1.51%) and ‘Black Monukka’ (1.41%) [18] but significantly higher than wild grapes (0.54%, *P* < 0.05) [29] (Fig. S1A and B). The assembled genomes, JA_hap1 (494.79 Mb) and JA_hap2 (494.13 Mb), were of high quality with BUSCO scores of 98.6% and 98.3%, and scaffold N50 values of 25.94 and 26.38 Mb, respectively, and additional metrics including quality value (QV) (50.3426 for hap1, 50.0721 for hap2; mean 50.2053), k-mer completeness (81.3577% for hap1, 81.5623% for hap2; combined 99.1256%), LTR assembly index (LAI) values (19.21 for hap1, 19.20 for hap2, both meeting ‘Reference’ classification), and a switch error rate of 1.19% (Table S2). Annotations revealed 19 centromeres and 35 telomeres (Table S2), and repeat masking covered ~333 Mb for each haplotype, consistent with typical *Vitis* genomes. GC content (35.07% and 34.98%) confirmed inter-haplotype consistency, while Hi-C data organized the assembly into 19 pseudo-chromosomes, validating structural integrity (Fig. 1D).

Comparative collinearity analysis with MH and PN40024 showed strong synteny, conserved genome sizes, and centromere positions, indicating evolutionary conservation (Fig. 1A). Between JA haplotypes, we identified 3671 hemizygous genes (~10.21% of all genes), consistent with other highly heterozygous grapevine genomes [30]. Gene ontology (GO) enrichment revealed these genes were involved in nitrogen metabolism, protein complex organization, and nucleic acid-related processes, suggesting roles in key cellular pathways (Fig. 1E).

### Pan-genomics and comparative genomics detected JA-specific SVs and genes

**Figure 2:**
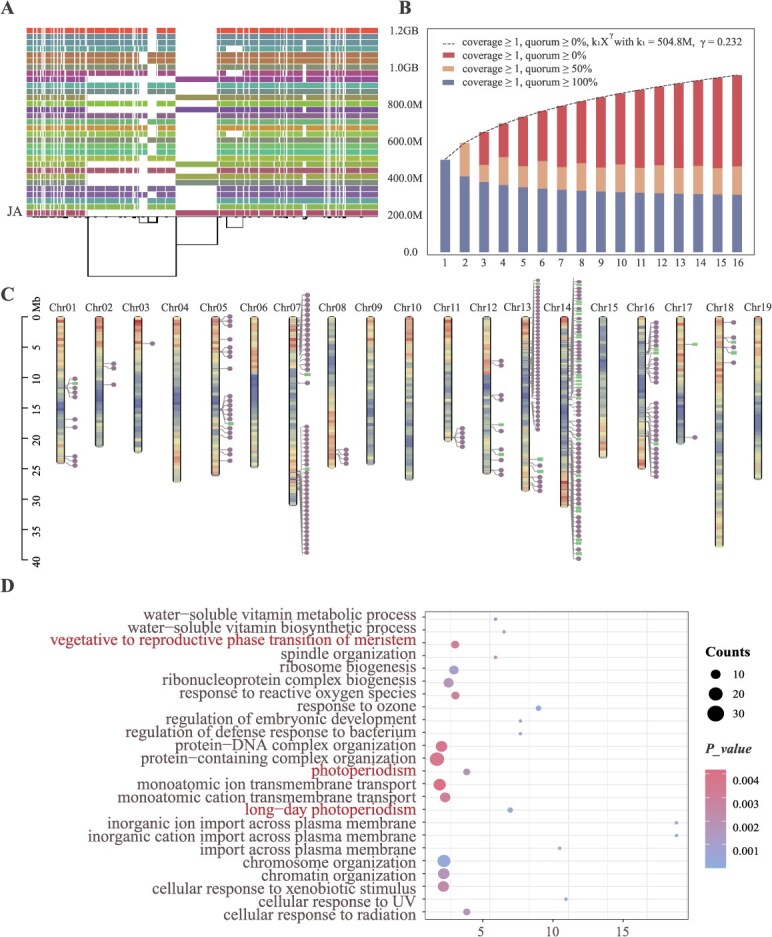
Pan-genomics identifies Julian-specific variations and genes in table grapes. (A) Schematic representation of the graph pan-genome constructed from 16 grape genomes, including 31 haplotypes. The graph illustrates structural variations such as insertions and deletions across different haplotypes, with the two JA haplotypes shown at the bottom. (B) Pan-genome growth curve depicting the increase in total pan-genome length as additional genomes are incorporated. (C) Distribution of JA-specific structural variants (unique SVs) on chromosomes, showing the first 50 insertions per chromosome for JA_hap1 genome. (D) GO enrichment analysis of genes located within 5 kb of JA unique SVs.

To investigate the genomic differences between the JA and other table grape cultivars, we constructed a pan-genome comprising the JA genome, 14 phased genome assemblies from other table grape cultivars, and the reference genome PN40024 [31]. The newly sequenced JA genome was integrated into this analysis to enable a comparative exploration of sequence variations. Using the Pan Genome Graph Builder (PGGB), we constructed a pan-genome graph (Fig. 2A), which spanned 987.06 Mb—approximately double the size of a single haploid grape genome (Fig. 2B). The pan-genome approach provided a comprehensive framework to capture sequence variations that linear genome models often overlook, including structural variants (SVs) and presence/absence variations (PAVs), both critical for understanding the genetic diversity underlying complex traits like continuous flowering.

The analysis identified 19 574 412 variants across the pan-genome, including 16 748 352 single nucleotide polymorphisms (SNPs), 2 643 897 insertions/deletions (indels), and 1 383 324 multi-nucleotide polymorphisms (MNPs). These variants highlight the extensive genetic divergence among table grape cultivars, likely driven by artificial selection and domestication processes [32]. Pan-genome-based studies in other crops, such as rice and sunflower, have similarly revealed that domestication often introduces unique structural changes and genetic diversity essential for agronomic traits [14, 15]. Such findings underscore the utility of the pan-genome approach in uncovering genetic variations linked to complex phenotypes.

By applying a 50 bp threshold to define SVs, we identified 8758 insertions and 2059 deletions, with 558 classified as unique JA-specific SVs (Table S6). Notably, chromosomes 7, 13, and 14 exhibited a concentration of these structural variants (Fig. 2C). Further analysis of genes within 5 kb of these variants revealed significant enrichment in pathways related to the transition from vegetative to reproductive growth, photoperiod regulation, and long-day photoperiod response (Fig. 2D).

### Single-cell transcriptome atlas of the grapevine bud

To investigate the molecular basis underlying continuous flowering in JA and compare it with MH, we conducted single-nucleus RNA sequencing (snRNA-seq) on both varieties. Gene expression analysis of grape buds from JA and MH revealed 13 482 high-quality single-nucleus transcriptomes from JA, covering 22 453 genes, with a median of 2067 genes and 3547 unique molecular identifiers (UMIs) per nucleus. For MH, 18762 transcriptomes representing 24 462 genes were obtained (Table S8). Notably, ~77% of the genes detected by snRNA-seq overlapped with those identified in bulk RNA-seq, highlighting the complementarity of these methods.

**Figure 3:**
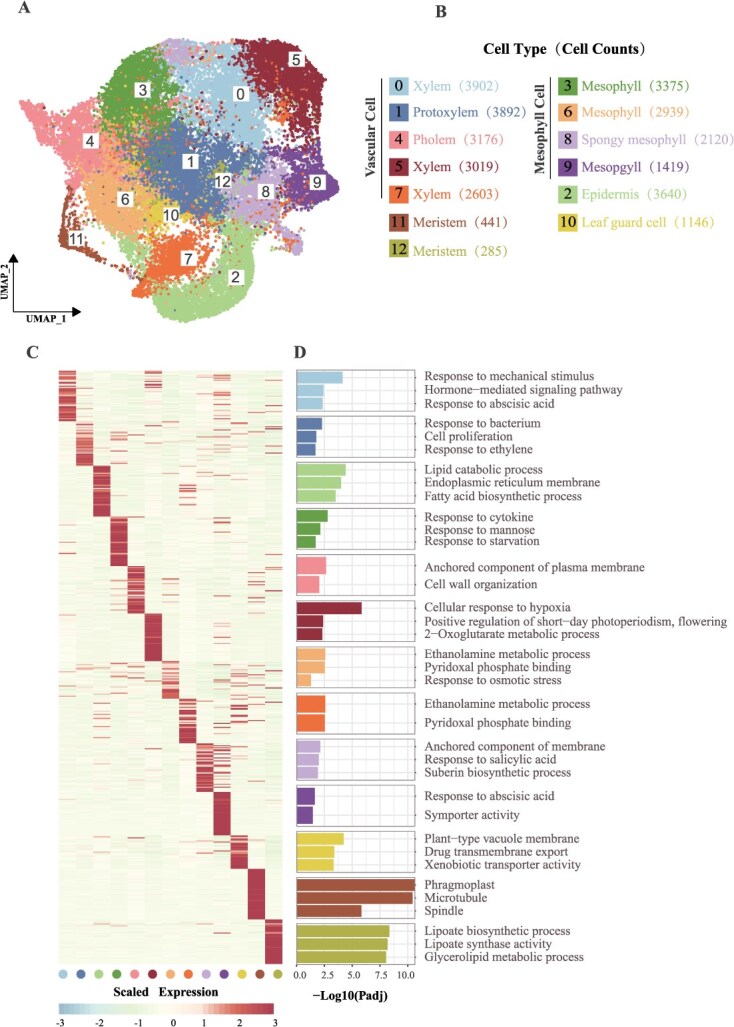
Single-nucleus transcriptome atlas of grapevine buds. (A) UMAP visualization of single-nucleus transcriptomes from JA and MH grape buds, revealing 13 distinct cell clusters. (B) Annotated cell types based on marker gene expression, with the number of nuclei indicated in parentheses. (C) Heatmap displaying the top 50 marker genes for each cell cluster, illustrating cell-type-specific gene expression. (D) GO enrichment analysis of the marker genes, highlighting significantly enriched biological processes associated with each cluster.

Graph-based clustering analysis in Seurat identified 13 distinct cell clusters, visualized using UMAP (Fig. 3A). Mapping homologous genes from *Arabidopsis thaliana* allowed us to assign these clusters to major cell types in grapevine buds (Fig. 3B, Table S10), including vascular cells (xylem and phloem), epidermal, mesophyll, and meristematic cells. Xylem cells (9524 cells) were most abundant, emphasizing their role in nutrient transport, followed by mesophyll cells (7733 cells), essential for photosynthesis. Notably, the detection of 726 meristematic cells is a key component in dormancy and flowering regulation.

After identifying the top 50 marker genes for each cell cluster (Table S11), we performed GO enrichment analysis, which revealed distinct functional enrichments specific to each cell type. Xylem cells were enriched in pathways related to water transport and cell wall modification, reflecting their structural and resource distribution roles. Mesophyll cells were strongly enriched in photosynthetic pathways, highlighting their function in energy production. In contrast, meristematic cells exhibited enrichments in cell division and developmental regulation pathways (Fig. 3C and D), indicating their potential role in dormancy and flowering regulation. These findings demonstrate that the marker genes identified for each cluster are closely tied to the specialized functions of grapevine bud cells, advancing our understanding of grapevine development and flowering.

### Differentially expressed genes (DEGs) in JA and MH

**Figure 4:**
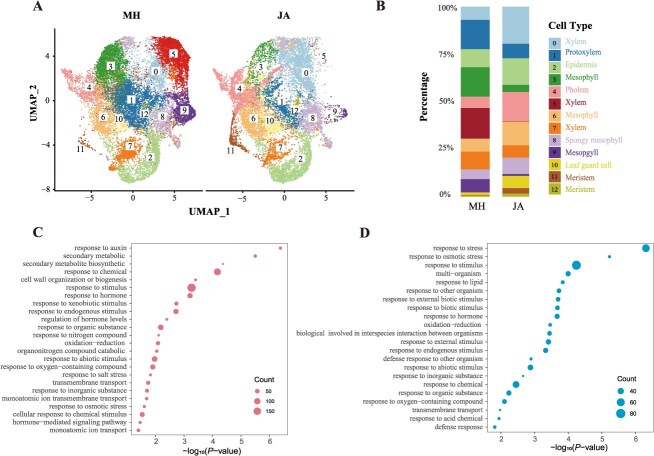
Differential expression and GO Enrichment in JA and MH grape buds. (A) UMAP visualization of cell clusters from snRNA-seq data in JA and MH, displaying the distribution and clustering of cells in each grape variety. (B) Comparative analysis of cell-type proportions between JA and MH, showing the relative abundance of different cell types in the grape bud samples. (C) GO enrichment analysis of DEGs upregulated in JA for biological processes. (D) GO enrichment analysis of DEGs upregulated in MH (downregulated in JA) for biological processes.

To further investigate the single-cell-level differences between JA and MH, we aligned their single-cell count matrices using the ‘anchor’ method (Fig. 4A), enabling a direct comparison of cell composition and gene expression between the two cultivars. This alignment revealed significant differences in cell type distributions, particularly in the abundance of meristematic and mesophyll cells (Fig. 4B). Subsequently, DEG analysis was performed on the snRNA-seq data from both JA and MH, resulting in the identification of 5807 DEGs (Fig. 4C, Table S12). Of these, 2919 genes were upregulated in JA, while 2888 genes were upregulated in MH. Among them (Table S12), key examples include Vitis18g01001, a *MED18* homolog regulating flowering time via *FLC* and *AG* in *Arabidopsi*s [33], significantly upregulated in Julian (5.2 TPM vs. 2.1 TPM in MH); and Vitis14g01538, an *AOC4* homolog critical for jasmonic acid biosynthesis, also upregulated in Julian, suggesting elevated jasmonic acid signaling [34]. Additional DEGs involve auxin/cytokinin pathway genes like Vitis07g00043(homologous to *ATAUX2–11*), collectively supporting Julian’s sustained flowering traits. To further explore the biological significance of these DEGs, we performed GO enrichment analysis, focusing on the upregulated genes in both JA and MH (Fig. 4D and E).

GO enrichment analysis of the upregulated genes in JA (Fig. 4D) showed significant enrichment in pathways related to growth, secondary metabolism, and hormonal regulation, including secondary cell wall biogenesis, phenylpropanoid biosynthesis, and auxin response. These pathways were associated with enhanced cell wall rigidity, tissue differentiation, and expansion, potentially supporting JA’s CFB. In MH, upregulated genes (downregulated in JA) were enriched in pathways such as carboxylic acid catabolism, lipid metabolism, and interspecies interactions (Fig. 4E), which are linked to metabolic regulation and responses to environmental factors. These differences in enrichment profiles indicate distinct molecular priorities in JA and MH, with JA emphasizing growth-related processes and MH showing stronger activation of stress-associated pathways.

### Integrative genomics of CFB in JA

**Figure 5:**
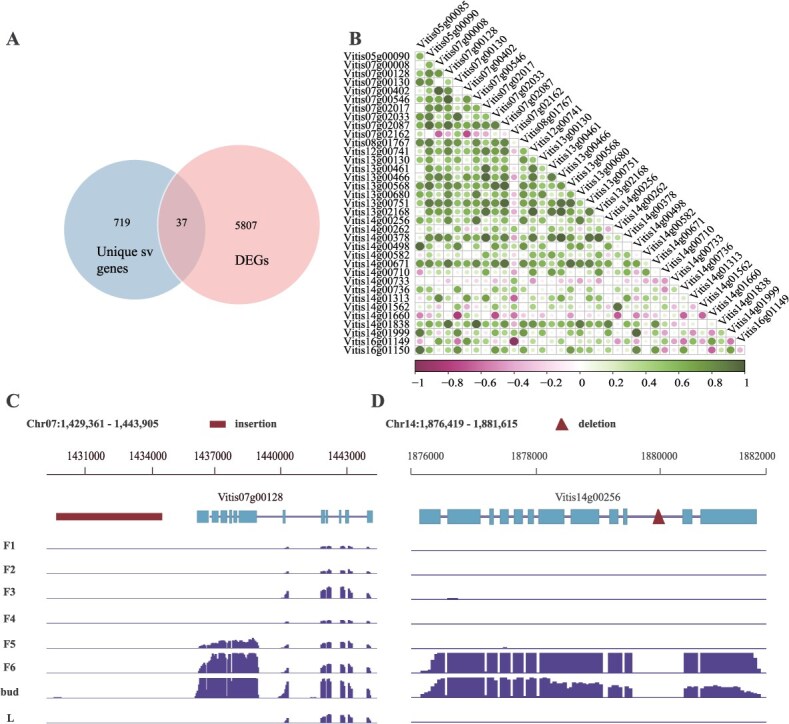
Analysis of candidate genes. (A) Venn diagram showing the overlap of genes from JA unique SVs, and DEGs identified in single-cell transcriptomics. (B) Correlation heatmap of candidate genes, circle size representing correlation strength. (C, D) Chromosomal location and expression patterns of candidate genes across fruit development stages (F1–F5), inflorescences (F6), leaves (L), and buds.

To uncover the genetic basis of the CFB in JA, we integrated SVs, genomic hotspots, and DEGs from single-cell transcriptomic data. This analysis identified 757 genes within unique SV regions (Table S7), which overlapped with 5807 DEGs, ultimately narrowing down to 37 candidate genes (Fig. 5A). Several candidate genes were implicated in pathways critical for rhythmic regulation, stress response, and cell wall modification, aligning with key traits observed in JA’s CFB phenotype. Correlation analysis of the expression patterns between candidate genes, as shown in Fig. 5B and detailed in Table S13, revealed several co-expression modules likely involved in shared regulatory pathways. For instance, Vitis07g00402 (pectinesterase) and Vitis07g00546 (GDSL esterase/lipase) displayed a strong correlation (0.964), suggesting a coordinated role in cell wall modification synchronized with circadian rhythms. This process may facilitate daily restructuring of the bud architecture, supporting sustained growth and development. Similarly, Vitis07g02033 (zinc finger protein ZAT10) and Vitis13g00130 (LOB domain-containing protein 12-like), with a correlation of 0.928, are involved in stress response and differentiation. ZAT10, known for integrating circadian modulation and stress pathways, may regulate bud development under environmental rhythms, contributing to JA’s adaptation to changing conditions. Vitis14g00733 (auxin-induced protein 22D) and Vitis14g00736 (pathogenesis-related protein PR-4), correlated at 0.678, are enriched in auxin signaling and defense pathways. Auxin’s circadian regulation suggests a synchronized role in tissue differentiation and growth maintenance, directly supporting continuous flowering.

Circadian-regulated stress and defense mechanisms were also evident in unique genes. Vitis07g00128, encoding a Vicilin-like antimicrobial peptide (*AMP*), harbors an 11 768 bp insertion upstream (Fig. 5C), potentially altering its expression. As a key player in defense and growth integration, its rhythmic expression aligns protective mechanisms with daily cycles, ensuring bud survival during vulnerable periods. Vitis14g00256 (respiratory burst oxidase homolog protein B, *RBOH*) features an intronic insertion and is highly expressed in buds and inflorescences during early development (Fig. 5D). Its circadian-regulated expression suggests a role in coordinating early bud growth and floral development, critical for maintaining JA’s CFB trait.

Further analysis identified gene pairs involved in lignin and phenolic biosynthesis, such as Vitis07g02162 and Vitis14g01562, which contribute to structural stability and bud hardening. These pathways are crucial for ensuring bud resilience and continuous flowering cycles. Additionally, photosynthesis-related genes such as Vitis14g00498 (BONZAI 3) and Vitis14g00582 (phytyl ester synthase 1), correlated at 0.954, highlight the integration of metabolic and rhythmic pathways in supporting energy demands during prolonged reproductive phases.

Our findings underscored the interplay of cell wall dynamics, hormonal regulation, and circadian control in JA unique CFB trait. The integration of SVs, DEGs, and functional annotations revealed a complex network of genetic regulation that enables JA to sustain flowering and fruiting cycles throughout the growing season.

## Discussion

Dissecting the genetic basis of CFB is crucial in plant biology and crop breeding, as these traits directly influence agricultural productivity and adaptability. However, the genetic basis of continuous flowering remains largely unexplored in crops. In our present study, we newly generated high-quality, haplotype-resolved genome assemblies for the grape cultivar JA, known for its CFB traits. By integrating multi-omics approaches, including pan-genomics, comparative genomics, single-cell, and bulk transcriptomics, we identified unique large SVs and DEGs that distinguish JA from control grapevines with concentrated flowering and fruiting periods. The study will help in understanding the genetic basis of continuous flowering trait in plants and provide a list of candidate unique SVs and genes for generating new grape varieties with continuous flowering.

### Pan-genomic and comparative genome analysis capture unique SVs and genes

Pan-genome and comparative genome analyses can discover the unique SVs or genes associated with important agronomic traits at unprecedented resolution. For instance, a pan-genome analysis of wild and cultivated soybean detected lineage-specific genes and copy number variations associated with flowering time and biotic resistance [35]. Similarly, in grapevine, pan-genome and comparative analyses have been employed to uncover SVs or genes tied to essential agronomic traits [16–18, 36]. The pan-genome of a specific grapevine variety identified SVs and genes related to berry and bunch size [16]. Another pan-genome grapevine study found that ~7% of genes were unique to each accession, contributing to traits such as aroma and disease resistance [17]. Furthermore, comparative genome analysis revealed large SVs that were crucial for the seedless trait [18]. In our study, we focused on the pan-genome of JA, a grapevine with continuous flowering capability, compared to other grapevines that flower in concentrated periods. This analysis identified a number of SVs unique to JA. GO analysis of 664 genes within these JA-unique SV regions showed enrichment in pathways associated with photoperiod regulation and flowering, such as long-day photoperiodism. These SVs are likely to represent conserved mechanisms crucial for flowering regulation, forming the genetic foundation for breeding programs focused on flowering traits.

Importantly, our pan-genome analysis identified genes specific to JA, including those affecting key genes, such as Vitis07g02034 (a light signal transduction protein) and Vitis14g00398 (a transcription factor related to the Nuclear Factor Y complex). The *NF-Y complex* is known to regulate flowering by influencing key genes like FLOWERING LOCUS T (*FT*) and *SOC1*, as demonstrated in Arabidopsis [37]. In addition, Vitis07g02159, encoding a SET domain-containing histone methyltransferase, and Vitis13g00556, a homolog of the regulator of nonsense transcripts, may contribute to the epigenetic regulation of flowering genes under specific light conditions, potentially allowing JA to maintain flowering activity across variable photoperiods. These structural variants provide a genomic basis for JA’s extended flowering capability, distinguishing it from other table grape varieties.

### Single-cell transcriptomics detect cell-type-specific gene expression

Single-cell transcriptomic approaches have greatly advanced our ability to study shoot apical meristems (SAMs) with unprecedented resolution, enabling the identification of cell types and transcriptional dynamics that were previously masked in bulk-tissue analyses [38]. For instance, in *Arabidopsis thaliana*, single-cell studies have highlighted the spatial organization and dynamic transitions of gene expression during embryogenesis and seed germination [28]. Single-nucleus RNA sequencing also provided high-resolution insights into cell-type-specific gene expression patterns in grapevine buds, a tissue rich in cellular heterogeneity and comprising various specialized cell types such as xylem, phloem, and meristematic cells [39]. In our study, 13 distinct cell clusters were identified, each contributing to nutrient transport, photosynthesis, or developmental regulation. Among these, meristematic cells play a pivotal role in sustained growth and organogenesis, as they house pluripotent stem cells that continuously generate new organs throughout a plant’s lifespan. GO enrichment analysis revealed that meristematic cells in JA were enriched in pathways associated with cell division, chromatin remodeling, and hormonal regulation, suggesting their critical contribution to the continuous flowering and bearing traits of JA.

Furthermore, to investigate the molecular basis of between JA (CFB) and MH (SFB), a comparative analysis of single-cell transcriptomes from both cultivars were conducted to focus on DEGs across various cell types, particularly in meristematic clusters. In JA, DEGs were significantly enriched in pathways related to cell division, chromatin remodeling, and hormone-mediated signaling processes. These findings suggested that JA’s meristematic cells maintain a higher level of transcriptional and epigenetic plasticity, which may support continuous organogenesis and floral initiation. For example, the upregulation of histone modification-related genes in JA could facilitate the sustained activation of flowering-related genes, enabling its prolonged reproductive activity. In contrast, MH displayed enrichment in stress-response and dormancy-related pathways, indicating a developmental strategy that prioritizes environmental resilience over continuous growth.

Auxin-responsive genes, which are critical for regulating cell differentiation and organ development, were prominently upregulated in JA’s meristematic cells compared to MH. This aligns with JA’s need for ongoing organogenesis to sustain its flowering phenotype. Similarly, DEGs in JA’s vascular tissues were enriched in pathways associated with nutrient transport and metabolic regulation, likely supporting the increased resource demands of continuous flowering and fruit production. Conversely, MH showed higher expression of genes involved in senescence and nutrient storage, reflecting its concentrated flowering strategy. These results highlight the distinct molecular landscapes of JA and MH, driven by differential transcriptional activity in key biological pathways. By linking DEGs to specific biological functions and cell types, our findings suggest that JA’s CFB trait is supported by enhanced transcriptional reprogramming and hormonal regulation, particularly within its meristematic cells.

### Integrative genomics dissect key genes associated with the circadian system

Integration of SVs, DEGs, and genomic hotspots led to the identification of 37 candidate genes potentially contributing to JA’s continuous flowering and bearing phenotype. Noteworthy genes include Zinc finger protein ZAT10 and members of the Cytochrome P450 family, as well as auxin-responsive and NAC domain-containing proteins, which are central to stress response and developmental processes [40–42]. These genes involvement in pathways such as GA-mediated cellulose synthesis, ethylene-regulated cell expansion, and ABA-controlled leaf senescence further underscored their potential regulatory roles in JA’s growth rhythms and development.

The genetic framework of continuous flowering extends beyond grapevines, as evidenced by parallels in species like tomato, rose, and strawberry. The TERMINATING FLOWER (*TMF*) gene in tomato [5], for instance, coordinates the flowering process, while genes like *VIL1*, *FRI3*, and *CO-like 2* in roses are linked to hormone-mediated regulatory mechanisms [43]. In strawberries, the SEASONAL FLOWERING LOCUS (*SFL*) enables perennial flowering irrespective of short-day conditions [44]. These cases illustrate the interplay between regulatory genes, hormone interactions, and environmental adaptability, providing a comparative framework for understanding continuous flowering across different plant species.

In conclusion, the integrated multi-omics approach employed in this research provides a robust framework for understanding the genetic underpinnings of continuous flowering in grapevines. Our findings not only enhance our knowledge of JA’s unique phenotype but also offer practical implications for grape breeding strategies. By leveraging insights from structural variants and gene expression patterns, this study paves the way for advancements in agricultural practices, aiming to optimize grape production, increase yield, and improve resource efficiency throughout the growing season. Future research should focus on functional validation of the identified candidate genes and exploration of their potential applications in breeding programs for other crops exhibiting continuous flowering traits.

## Materials and methods

### Plant materials and genome sequencing

The JA grapes were cultivated in the vineyard of Qingdao Agricultural University. Young leaves were collected for genome sequencing. Additionally, buds from the same nodes of both JA and MH grapevines were harvested for RNA sequencing. For RNA-seq, samples were collected from JA at five developmental stages along the same branch, from mature berries to inflorescences, as well as from leaf samples, with three biological replicates for each stage. All collected samples were rapidly frozen in liquid nitrogen prior to sequencing. Genome sequencing of JA was performed using PacBio HiFi long-read sequencing and Hi-C technology, both on a third-generation sequencing platform.

### Genome assembly and annotation

Before assembly, a preliminary genome assessment was conducted. A k-mer histogram was generated using Jellyfish (v2.3.0) [45] with the parameters -C, -m 21, -s 1000000000 to compute k-mer frequencies from sequencing reads with a k-mer size of 21. The k-mer frequency distribution was subsequently visualized and analyzed using GenomeScope (v2.0) [46] to infer genome characteristics, including genome size and heterozygosity rate.

First, raw Hi-C data were subjected to quality control and filtering using fastp (v0.23.2) [47], which removed low-quality reads and adapter contamination (Table S1). Hifiasm (v0.15.5) [48] was used to assemble and phase the diploid genome into preliminary haplotypes. Hi-C reads were aligned to contigs using Juicer (v2.0) [49], enabling chromosome anchoring and size calculation. Additionally, RagTag (v2.1.0) [50], with default parameters, aligned and oriented contigs to the Cabernet Sauvignon reference genome, forming scaffolds. The scaffolds were then refined at the chromosome level using 3D-DNA (v201008) [51] to detect and correct potential mistakes. Manual curation of scaffolds was performed in Juicebox [52] to ensure assembly accuracy. The final scaffold-level assembly was completed using 3D-DNA, resulting in two phased haplotypes with minimal gaps.

Next, genome quality metrics, including genome length, N50, and GC content, were calculated using seqkit (v2.2.0) [53]. Genome completeness was assessed using BUSCO (v5.3.0) [54] with the embryophyta_odb10 database. Additionally, we evaluated additional assembly quality metrics using specialized tools: LAI (longest anchor interval) values were calculated by first identifying LTR sequences with LTR_FINDER [55] and then analyzing these sequences with LTR_retriever [56] to derive LAI scores; QV (quality value) and k-mer completeness values were computed using Merqury (v1.3.1) [57] to assess sequence accuracy and continuity; and switch error rates, which reflect long-read alignment consistency, were determined using the calc_switchErr pipeline [58] (https://github.com/tangerzhang/calc_switchErr).

The gene structure and repeat sequences (Table S4) of the JA genome were annotated using a genome-wide annotation pipeline (https://github.com/unavailable-2374/Genome-Wide-Annotation-Pipeline). Additionally, an annotation file was generated using Liftoff (v1.6.3) [59] by mapping the PN40024 (V.5) reference genome to the JA genome.

To locate centromeres, we used trf (v4.09) [60] to examine the length and distribution of tandem repeat monomers throughout the genome, discovering a continuous and frequent occurrence of 107 bp monomers in centromeric regions (Table S3). The centromeric regions were then defined by integrating TRF findings with TE annotations. For telomeric repeat identification, we employed the Telomere Identification Toolkit (v0.2.0) [53] to detect instances of the 6 bp motif (TTAGGG) within the genomic sequence.

### Synteny analysis and identification of hemizygous genes

The entire genomes of JA, MH and PN40024 were aligned using Nucmer (v4.0.0) [61] with the parameters —maxmatch, -c 100, -b 500, and -l 50 to generate pairwise alignments. Alignment outputs in delta format were filtered with Delta-filter using the parameters -m, -i 90, and -l 100 to retain alignments with high identity and alignment length. Filtered alignments were converted to coordinate format using show-coords -THrd for further downstream analysis. Structural variations (SVs), including inversions, translocations, and duplications, were identified and analyzed using Syri (v1.6.4) [62], which processed filtered delta files and coordinates with the parameter -F T to detect both collinear regions and non-collinear events across the genomes (Table S5). The results were visualized using Plotsr (v1.0.1) [63].

To identify hemizygous genes, we first mapped HiFi reads to the JA haplotype genome using Minimap2 (v2.24) [64] and sorted the resulting BAM files with samtools (v1.15.1) [65]. Structural variants were detected using Sniffles (v2.0.6) [66] with default parameters. Variants were filtered based on the following criteria: DEL type, END present, precise breakpoints, length ≥ 50 bp, quality ≥60, and support ≥4 reads [30]. Variants meeting these criteria were further analyzed, and those overlapping entirely with gene regions were classified as hemizygous genes.

### Pan-genome construction and variant calling

To construct a pan-genome graph, 15 haplotype-assembled grape genomes, including the haploid PN40024 and both haplotypes from 14 table grape varieties, were used. These genomes were specifically chosen to represent cultivated table grapes characterized by high levels of diploid heterozygosity. To ensure consistency and reliability in the pan-genome construction, all selected genomes were generated using third-generation sequencing technologies and assembled with a unified methodology, which collectively contributed to their high-quality assembly outcomes. To ensure consistency across datasets, all haplotypes were uniformly named using the format Name#Haplotype#Chromosome (e.g., JA#1#chr01 for chromosome 1 of haplotype 1 in JA). All sequences were merged into a single FASTA file for input. The initial graph construction was performed with PGGB (v0.6.0) [67] using the parameters -s 5000, -l 25 000, -p 90, -c 1, -K 19, -F 0.001, -g 30, -k 23, -f 0, and -B 10 M, generating partitioned chromosome-specific files based on genomic community structure. The final GFA file was obtained by running commands extracted from PGGB’s log file. For structural variation (SV) calling, vg deconstruct was used with JA as the reference genome to produce a VCF file. We first extracted rows from the merged VCF file where all genotype values were non-zero and not missing (.|.). From these, rows with all genotype calls consistently 1|1 were further identified and defined as unique-SV, representing loci with uniform homozygous alternate allele calls across genomes. After graph construction, Panacus (v0.0.2) [68] was used to calculate pan-genome growth, pan-genome size, and core genome size. Following SV calling, bcftools (v1.13) [65] view was employed to analyze the VCF file, extracting and statistically analyzing SNPs, indels, and MNPs. Additionally, a custom Python script was developed to determine the type of unique SVs: if the reference length was greater than the query length, the SV was classified as an insertion (Fig. S2B); otherwise, it was classified as a deletion (Fig. S2A).

### 10× snRNA-seq library construction and processing

Nuclei were isolated using a previously published method [69], with ~20 000 nuclei loaded into the 10× Genomics Chromium Controller to generate single-nucleus RNA-seq libraries. The nuclei were then encapsulated with reagents and barcoded gel beads in oil droplets using a microfluidic chip to form Gel Beads in Emulsions (GEMs), where RNA was released and reverse transcribed. The resulting cDNA was recovered, amplified, and subjected to quality control, followed by library construction. Sequencing was performed on the Illumina NovaSeq 6000 platform with 150 bp paired-end reads, generating ~100 GB of clean data per sample, across two biological replicates, totaling four samples and 400 GB of clean data.

The snRNA-seq data were processed using 10× Genomics Cell Ranger (v7.1.0) [70] to generate gene-by-cell matrices from demultiplexed FASTQ files. First, a custom reference genome for JA nuclear transcriptome was built using cellranger mkref function, specifying the genome sequence and annotation files. This custom reference was then used to align reads from each biological replicate of JA and MH using the cellranger count function. For each sample, alignment was performed with the ‘—include-introns’ flag enabled (Table S8). The resulting outputs for two biological replicates per cultivar were aggregated using the cellranger aggr function, with the *—normalize = mapped* parameter to normalize read depths across replicate samples before calculating cell-type proportions. (Table S9, Fig. S3A and B). The aggregated gene-by-cell matrices were subjected to downstream analysis using Seurat (v5.1.0) [71]. Quality control criteria were applied to retain nuclei with detectable gene counts between 200 and 7500 and fewer than 15 000 unique molecular identifier (UMI) counts (Fig. S3C and D). The resulting high-quality feature matrix was used for further clustering, dimensionality reduction, and visualization of cell populations. By integrating data across biological replicates, we ensured robust and reproducible analyses of gene expression patterns.

### Cell clustering, marker gene identification and differential expression analysis

To integrate the single-cell transcriptome data from the JA and MH samples, we first identified highly variable genes across both datasets using the ‘SelectIntegrationFeatures’ function in Seurat. The top 3000 genes with the most significant variability were selected as features for integration. These genes were then used to identify common features between the datasets through the ‘FindIntegrationAnchors’ method, which calculates integration anchors. The integration process was then carried out using ‘IntegrateData (normalization.method = “LogNormalize”)’ ensuring that data from both samples could be combined effectively while preserving biological signals and minimizing technical variability.

After integration, dimensionality reduction and clustering were performed to identify distinct cell populations. To determine the optimal number of principal components (PCs) for downstream analysis, a quantitative approach was employed. First, the proportion of variance explained by each PC was calculated, and the cumulative variance was computed to assess the contribution of the PCs collectively. Two criteria were applied to identify the optimal PC cutoff: (i) the cumulative variance explained exceeded 90%, and (ii) the individual variance explained by a PC was less than 5%. Additionally, the differences in variance explained between consecutive PCs were analyzed to capture the point where the variance drop stabilized. To validate this selection, an elbow plot was generated, displaying the variance explained by each PC. A vertical line was used to mark the selected cutoff point, corresponding to the optimal number of PCs. Based on this evaluation, 13 PCs were selected for downstream analysis (Fig. S4A). Dimensionality reduction was performed using Uniform Manifold Approximation and Projection (UMAP) with the selected PCs, enabling visualization of cell populations. Clustering was achieved using the FindNeighbors function with dimensions set to 1:13, followed by the FindClusters function at a resolution parameter of 0.4, resulting in distinct cell clusters. Marker genes for each cluster were identified using the FindAllMarkers function with the parameters only.pos = TRUE, min.pct = 0.25, and logfc.threshold = 0.25. These settings ensured that only positively enriched marker genes, expressed in at least 25% of cells within a cluster, were reported. The top 10, 20, and 50 marker genes for each cluster were ranked by average log fold change (avg_log2FC) and exported as CSV files for further analysis (Tables S11 and S12). Heatmaps of the top 10, 20, and 50 marker genes were generated to visualize their expression patterns across clusters, highlighting the distinct transcriptional profiles of each population.

To annotate cell types, cluster-specific marker genes were compared with previously reported marker genes from *Arabidopsis thaliana* through orthologous gene comparisons. Protein sequences for A. thaliana were downloaded from the TAIR database [72] and aligned to grapevine protein sequences (Table S10). Marker gene identification was facilitated using the scPlant [73]. Annotation of clusters was further supported by GO enrichment analysis performed on the top 50 marker genes from each cluster (Table S11). For each cluster, two representative marker genes were selected, and their correlation was visualized using custom R scripts (Figs S5–S7).

Cluster-specific marker genes were subjected to differential expression analysis between JA and MH samples. Metadata in the Seurat object was updated to include sample group information, categorizing cells into JA or MH groups based on their orig.ident labels. For each cluster, a subset of the Seurat object corresponding to that cluster was created, and the FindMarkers function was used to identify DEGs between JA and MH samples within each cluster. The parameters min.pct and logfc.threshold were kept at their default values of 0.1 and 0.25, respectively, ensuring the identification of biologically meaningful DEGs. The DEG results for all clusters were consolidated into a single data frame and annotated with cluster identifiers. This comprehensive DEG dataset was exported as a CSV file for downstream analyses (Table S12). Heatmaps of DEGs were generated to visualize expression differences between JA and MH across all clusters.

### RNA-Seq analysis and gene expression correlation

RNA-Seq data from JA grapevine buds at developmental stages F1–F5 were aligned to the JA haplotype 1 genome reference using HISAT2 (v2.2.1) [74] with paired-end mode enabled (-q -x), specifying the genome index and the paired read files (−1 and − 2). The alignment outputs in SAM format were converted to BAM, sorted, and indexed using samtools (v1.10) [65] with the view -bS, sort, and index commands. Gene-level counts were then generated using featureCounts (v2.0.4) [75] with the parameters -a for the GTF annotation file, -p for paired-end reads, -g gene_id to group counts by gene ID, and -t exon to target exon-level counts. For expression quantification, raw counts were normalized to Transcripts Per Million (TPM) values (Table S13). For correlation analysis, TPM data for candidate genes were filtered to exclude rows with zero values, and Spearman’s rank correlation coefficient was calculated to assess co-expression patterns among genes (Table S14). Correlation matrices were generated and visualized with the R package using the parameters order = ‘hclust’ and method = ‘color’, applying hierarchical clustering to reveal gene expression relationships, and output for further analysis.

## Supplementary Material

Web_Material_uhaf228

## Data Availability

The raw sequencing data, comprising PacBio HiFi long-reads, Illumina Hi-C reads, RNA-seq reads, and snRNA-seq reads, is accessible on NCBI under BioProject ID PRJNA192950 and on the National Genomics Data Center (NGDC) under BioProject ID PRJCA033121. The genome assembly and their annotations have been deposited into in Zenodo:10.5281/zenodo.14263262.
