# Supplementary material for: Pangenomics and single-cell transcriptomics uncover the genetic basis of continuous bearing trait in grapevine
Source: Hortic Res. 2025 Sep 2;12(12):uhaf228. doi: 10.1093/hr/uhaf228 (PMC12680498; doi:10.1093/hr/uhaf228)
Supplement: Web_Material_uhaf228 [file web_material_uhaf228.zip › Extended Data Fig.docx]

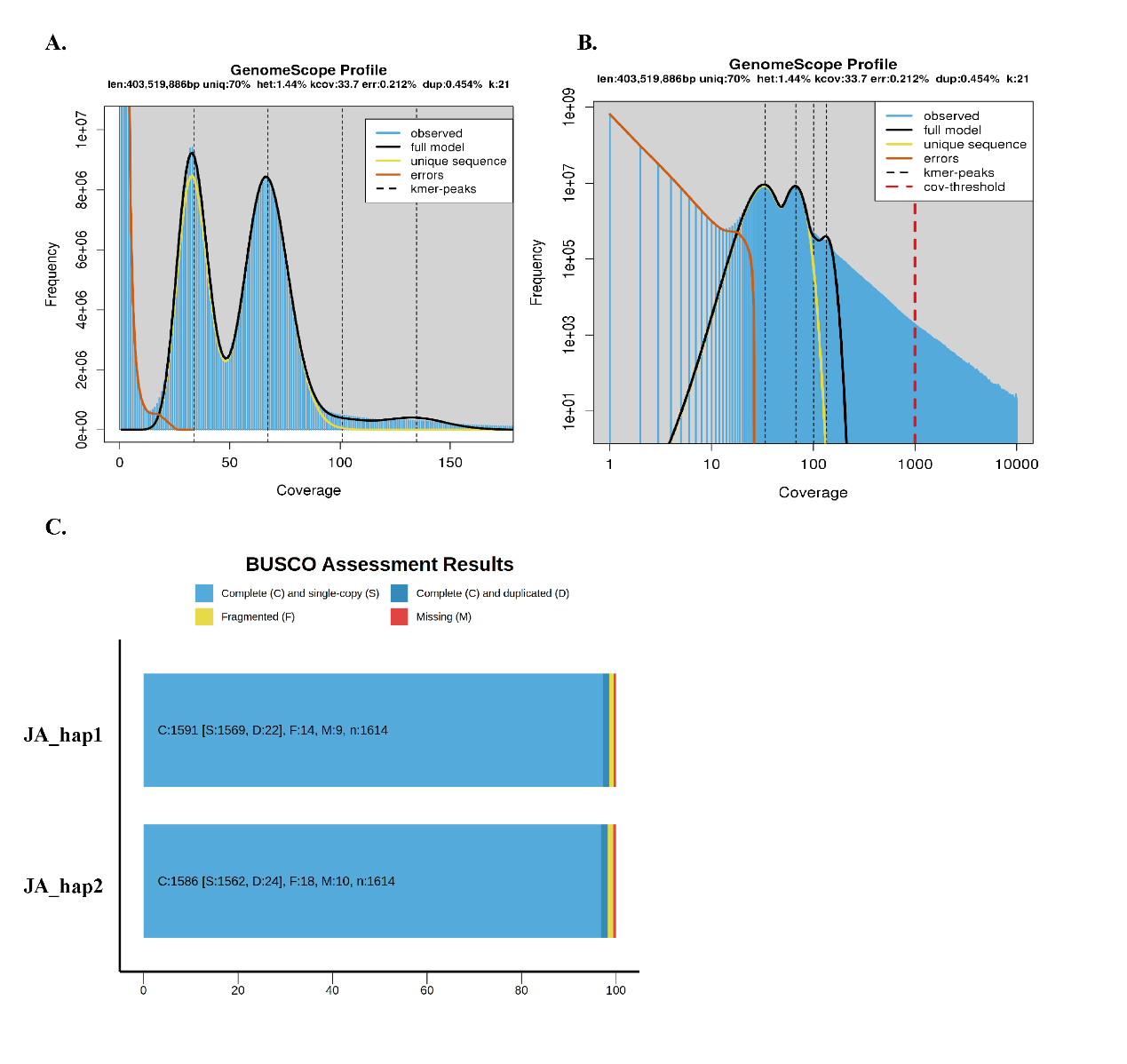


**Extended Data Fig. 1 | Pre-assembly assessment and quality evaluation of the haplotype-resolved genome assembly.** A, B, Pre-assembly evaluation results using k-mer analysis (k-mer length of 21) to assess genome size and heterozygosity. C, BUSCO assessment of genome completeness for the assembled haplotypes using the embryophyta_odb10 database.


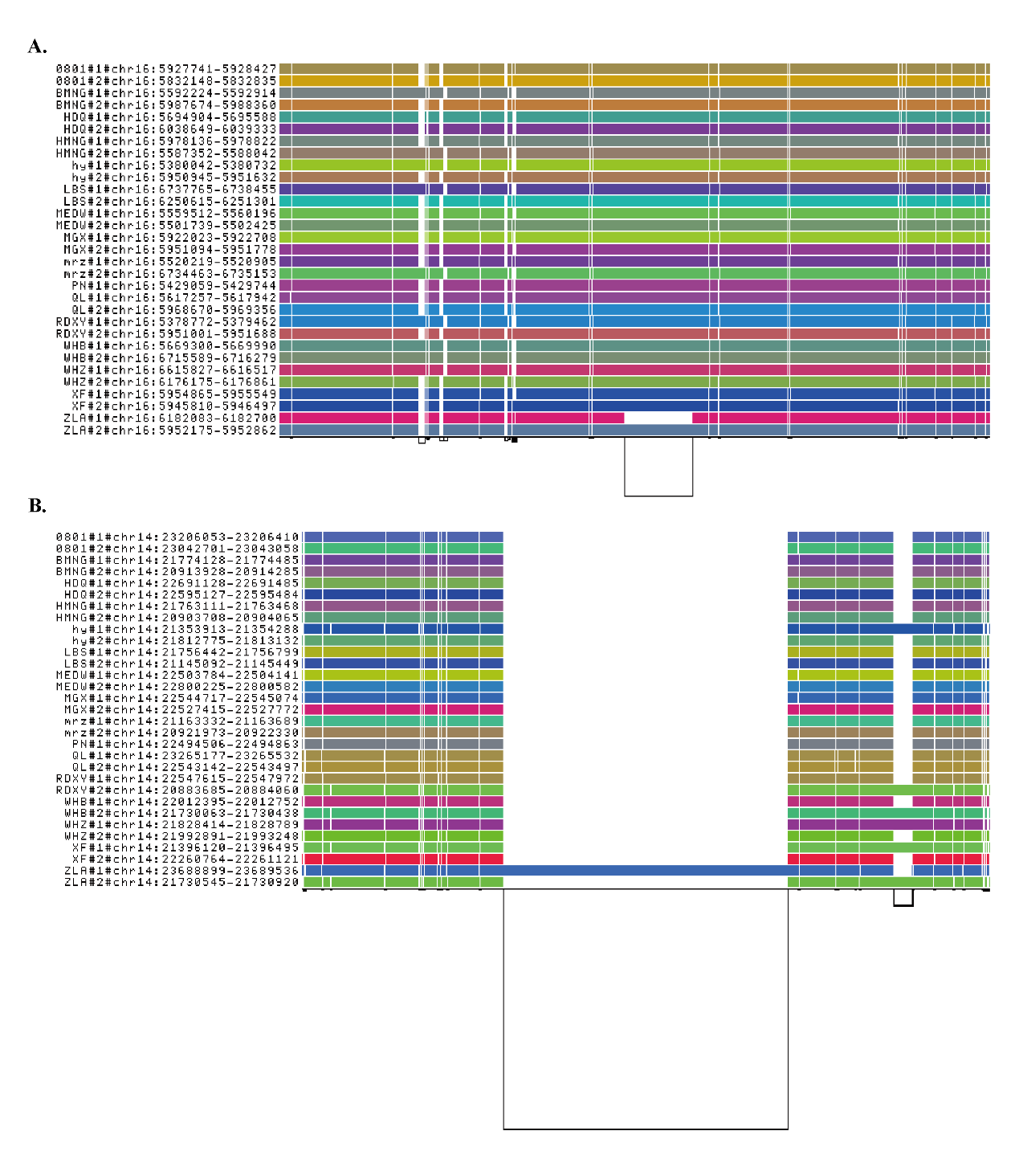


**Extended Data Fig. 2 | Comparative genomics of the pangenome to identify JA-specific variations.** A, Identification of a unique 70 bp deletion on chromosome 16 of JA haplotype 1 (ZLA#1#) through pangenome analysis. B, Detection of a 281 bp JA-specific insertion on chromosome 14 of JA haplotype 1.


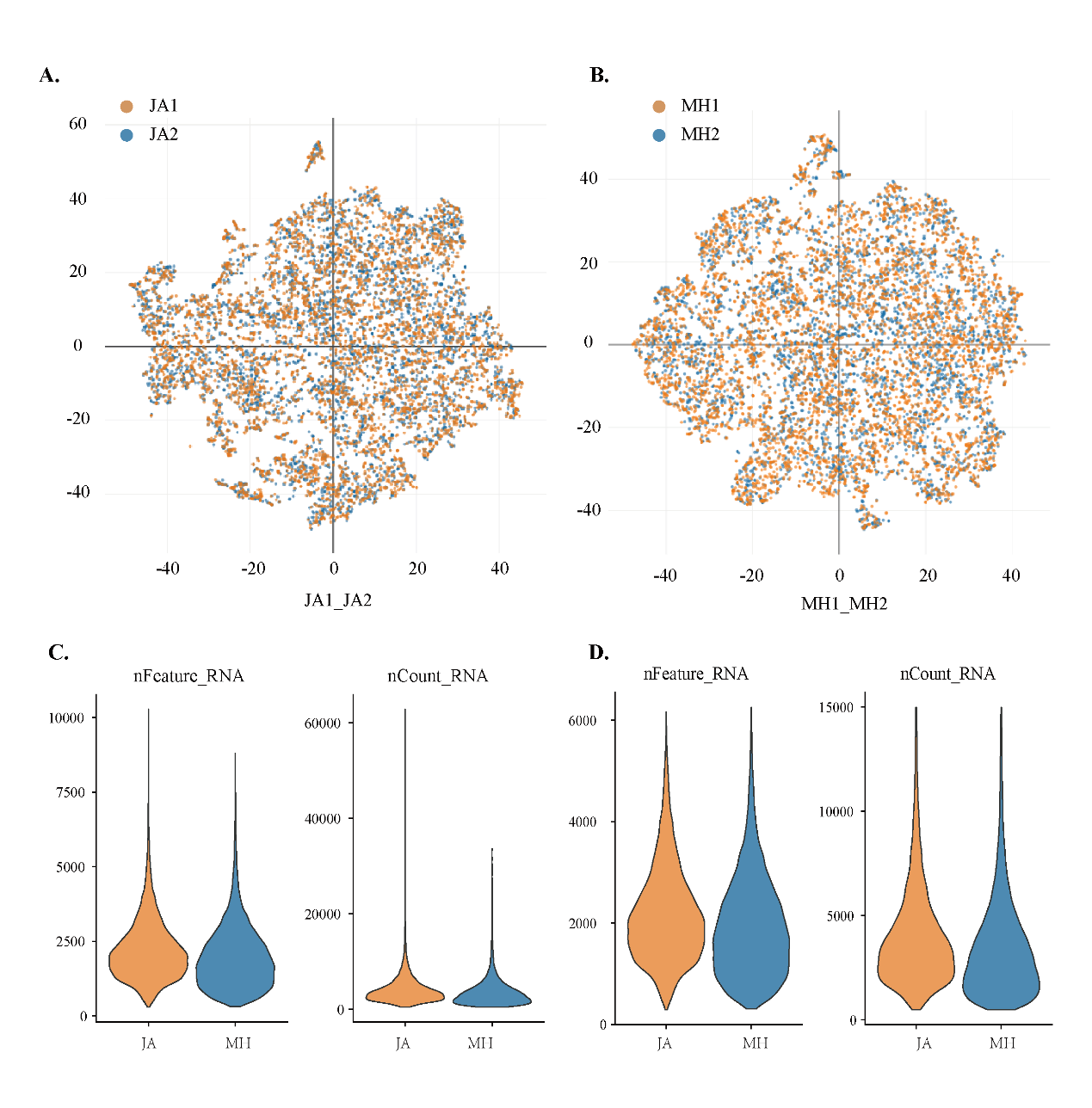


**Extended Data Fig. 3 | Pre-processing of single-nucleus transcriptome data.** A, t-SNE projection of JA samples, showing data distribution across two replicates, with each color representing a different replicate. B, t-SNE projection of MH samples, displaying the data distribution of its two replicates, with colors indicating each replicate. C, Distribution of feature and count before data filtering, with different colors representing JA and MH samples. D, Distribution of feature and count after data filtering, showing the cleaned data set.


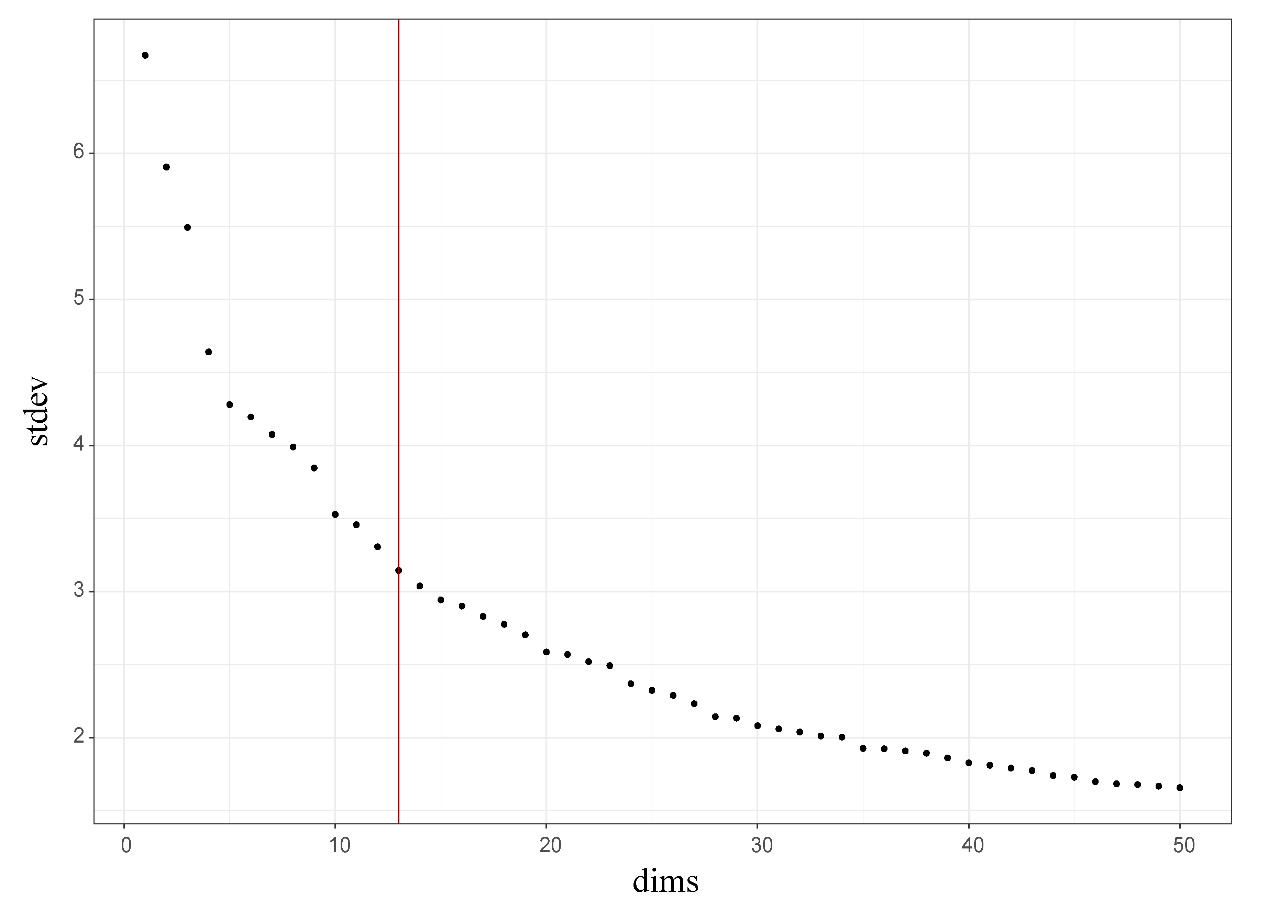


**Extended Data Fig. 4 | Elbow plot for selecting the optimal number of principal components (PCs).** The variance explained by each PC is shown, with a red line indicating the cutoff at 13 PCs, selected based on a cumulative variance exceeding 90% and an individual variance below 5%.


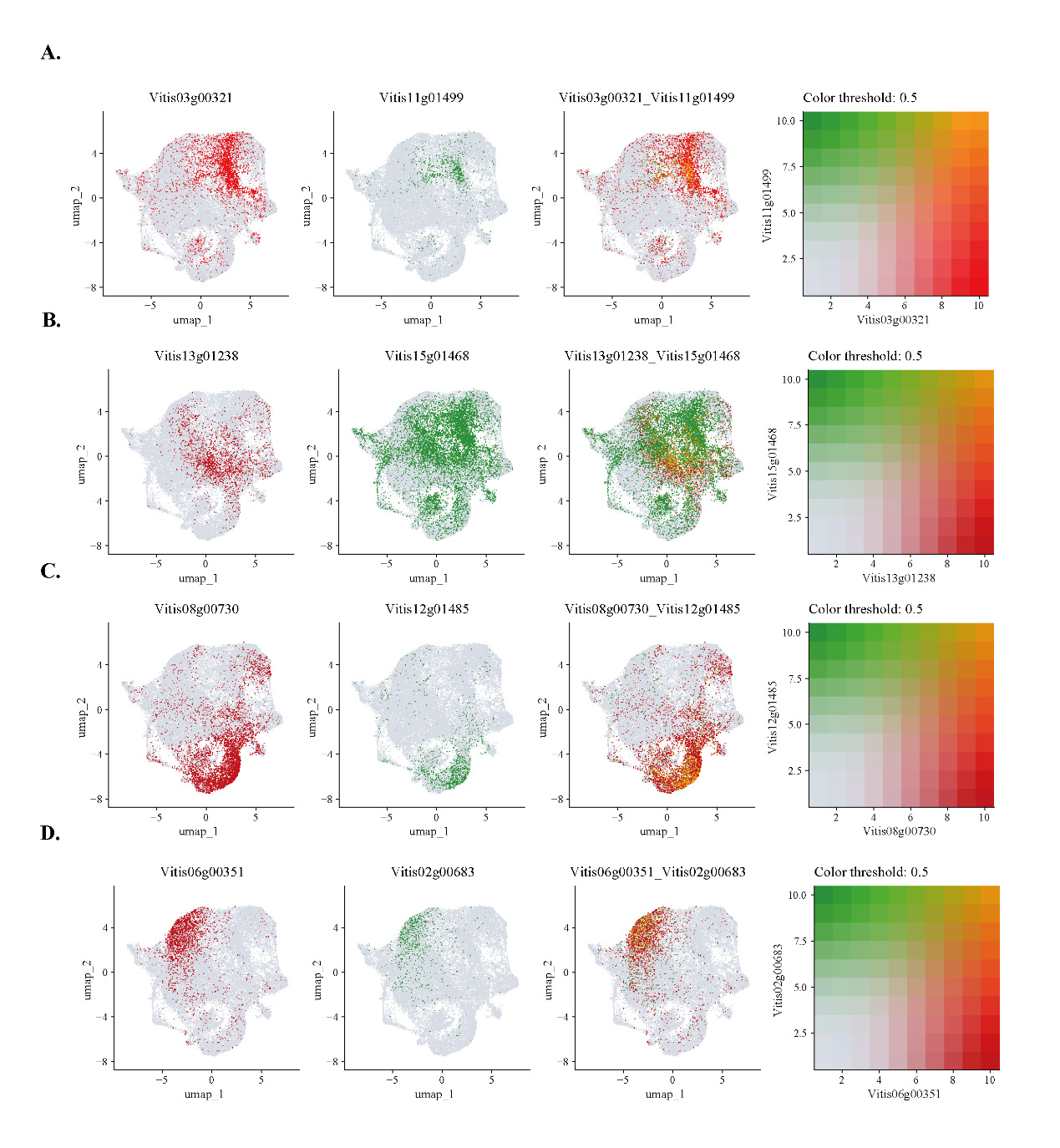


**Extended Data Fig. 5 | UMAP distribution of marker genes across clusters in single-nucleus transcriptome data.** Each panel displays the spatial distribution of two selected marker genes within individual clusters on UMAP plots. (A–D) illustrates clusters 0–3, red and green indicate the expression of individual marker genes, while yellow highlights regions of high co-expression within each cluster.


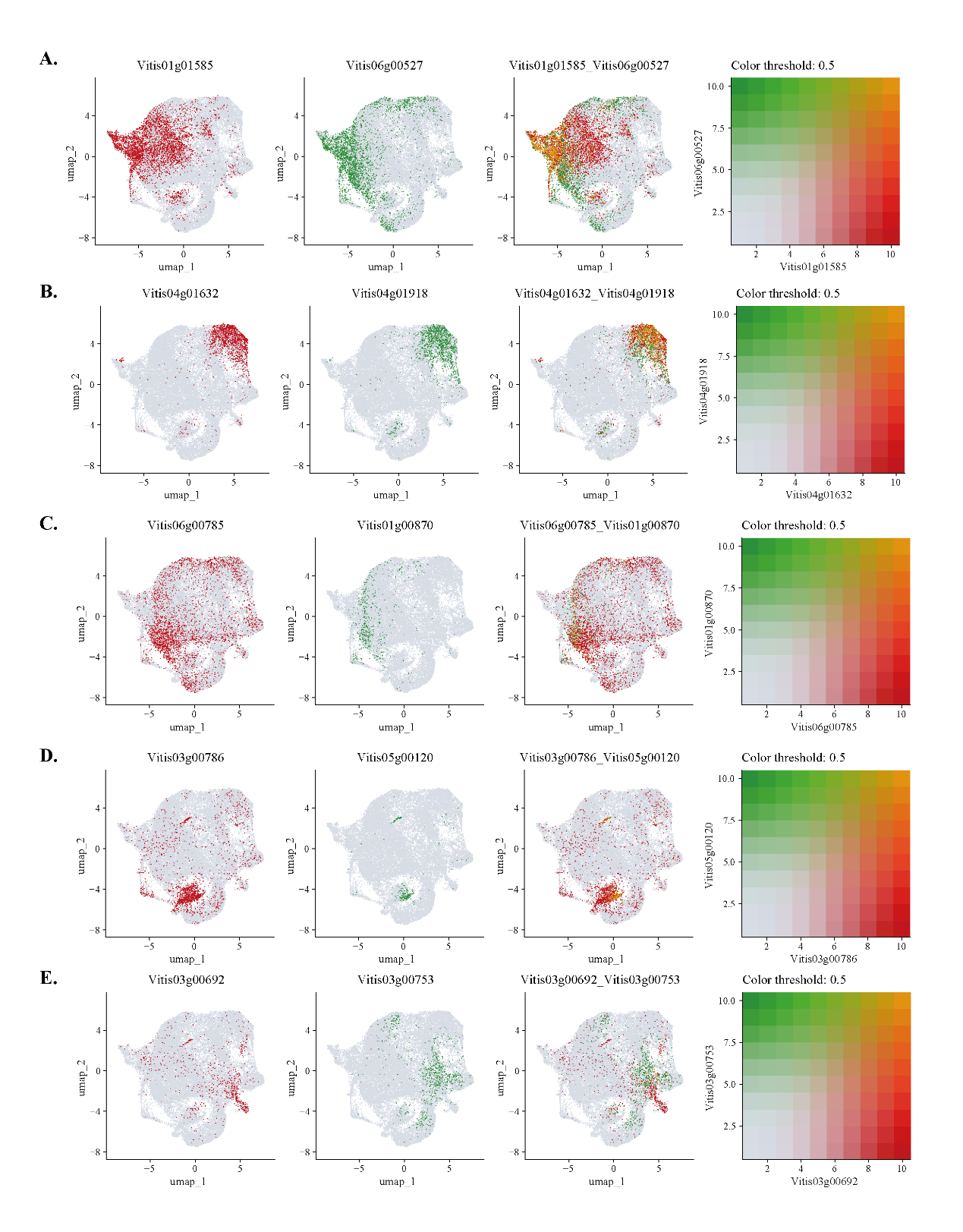


**Extended Data Fig. 6 | UMAP distribution of marker genes across clusters in single-nucleus transcriptome data.** Each panel displays the spatial distribution of two selected marker genes within individual clusters on UMAP plots. (A–E) covers clusters 4–8, red and green indicate the expression of individual marker genes, while yellow highlights regions of high co-expression within each cluster.


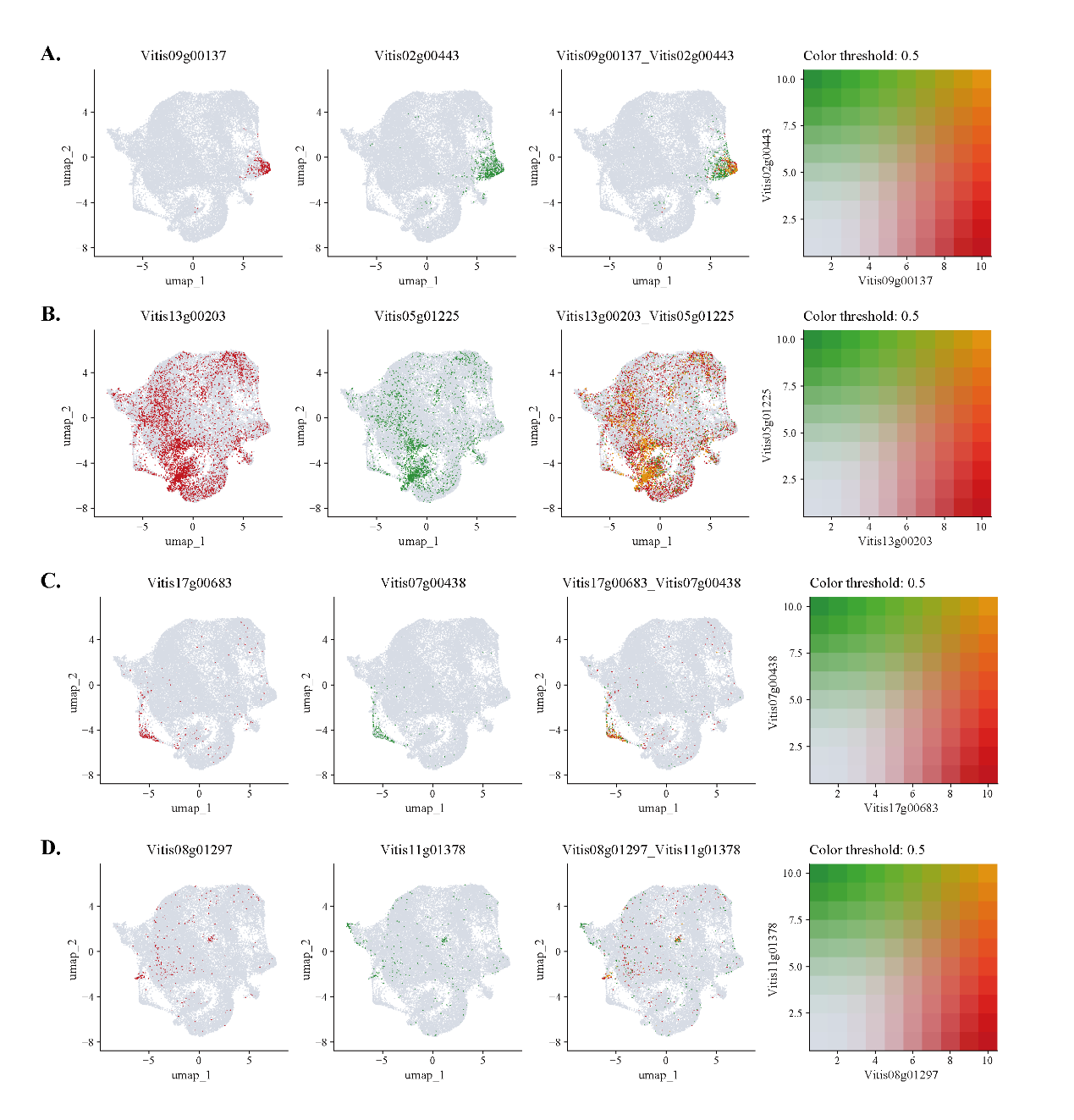


**Extended Data Fig. 7 | UMAP distribution of marker genes across clusters in single-nucleus transcriptome data.** Each panel displays the spatial distribution of two selected marker genes within individual clusters on UMAP plots. (A–D) represents clusters 9–12, red and green indicate the expression of individual marker genes, while yellow highlights regions of high co-expression within each cluster.
